# Biological significance and prognostic/predictive impact of complex karyotype in chronic lymphocytic leukemia

**DOI:** 10.18632/oncotarget.26146

**Published:** 2018-09-28

**Authors:** Maurizio Cavallari, Francesco Cavazzini, Antonella Bardi, Eleonora Volta, Aurora Melandri, Elisa Tammiso, Elena Saccenti, Enrico Lista, Francesca Maria Quaglia, Antonio Urso, Michele Laudisi, Elisa Menotti, Luca Formigaro, Melissa Dabusti, Maria Ciccone, Paolo Tomasi, Massimo Negrini, Antonio Cuneo, Gian Matteo Rigolin

**Affiliations:** ^1^ Hematology Section, Department of Medical Sciences, Azienda Ospedaliero-Universitaria, Arcispedale S. Anna, University of Ferrara, Ferrara, Italy; ^2^ Department of Morphology, Surgery and Experimental Medicine, University of Ferrara, Ferrara, Italy

**Keywords:** chronic lymphocytic leukemia, complex karyotype, prognosis, target therapy, Richter transformation

## Abstract

The complex karyotype (CK) is an established negative prognostic marker in a number of haematological malignancies. After the introduction of effective mitogens, a growing body of evidence has suggested that the presence of 3 or more aberrations by conventional banding analysis (CBA) is associated with an unfavorable outcome in chronic lymphocytic leukemia (CLL). Thus, the importance of CBA was recognized by the 2018 guidelines of the International Workshop on CLL, which proposed the introduction of CBA in clinical trials to validate the value of karyotype aberrations.

Indeed, a number of observational studies showed that cytogenetic aberrations and, particularly, the CK may have a negative independent impact on objective outcome measures (i.e. time to first treatment, progression free survival, time to chemorefractoriness and overall survival) both in patients treated with chemoimmunotherapy and, possibly, in patients receiving novel mechanism-based treatment.

Here, we set out to present the scientific evidence supporting the significance of CK as a prognostic marker in CLL and to discuss the biological basis showing that the CK is a consequence of genomic instability.

## INTRODUCTION

Chronic lymphocytic leukemia (CLL) is the most common leukemia of the adult in western countries [1–2]. As a result of genetic and biological complexity [3], the disease runs a variable clinical course, with some patients showing a very indolent evolution and others rapid progression [4]. Several biological features were identified which may predict the time between the diagnosis and the disease progression, the overall survival (OS) (i.e. prognostic markers) and the quality of response to a specific agent (i.e. predictive markers) [5]. The mutational status of the variable portion of the immunoglobulin gene (*IGHV*) [6], chromosome 17p deletion as detected by fluorescence *in situ* hybridization (FISH) [7] and *TP53* gene mutations [8] represent powerful prognostic/predictive factors commonly used to stratify CLL patients into different risk groups in the era of chemoimmunotherapy [5, 9–10]. The introduction of effective mechanism-based treatment (i.e. ibrutinib, idelalisib, venetoclax) significantly improved the outcome of CLL [11–13] and the adverse impact of 17p-/*TP53* mutation and of the *IGHV* mutational status were found to be attenuated in several studies [14–16].

Cytogenetic abnormalities in CLL were first described using chromosome banding analysis (CBA) in the 1970s [17] and in 1990 the complex karyotype (CK) was found to be associated with shorter survival [18]. At that time conventional cytogenetics was limited by the low number of dividing cells in culture [19]. Since 2006, the introduction of the effective mitogens CpG-oligodeoxynucleotide (DSP30) and Interleukin (IL)-2, dramatically improved the mitotic yield [20–21] and 65-83% of CLL patients were shown to carry a karyotype aberration [22–24]. Furthermore, evidence was provided that 21.5-35.7% of CLL cases without aberration by FISH carried chromosome aberrations by CBA, including CK, that were associated with an inferior outcome [25]. Overall, a CK is detectable in 14-34% of untreated CLL patients [23–24, 26–29], and up to 25-35% in the relapse refractory setting [30].

The CK has a strong adverse prognostic significance in several hemopoietic neoplasms such as acute myeloid leukemias, myelodysplastic syndromes and myelofibrosis [31–36] and a number of papers were published in the last 10 years highlighting its relevance in the landscape of prognostic biomarkers in CLL. We therefore set out to review here the biological basis underlying the development of CK and the prognostic and predictive value of this cytogenetic pattern in CLL in the chemoimmunotherapy and the mechanism-based treatment era.

## METHODS

In this analysis the CK was defined by the presence of at least three clonal aberrations in the same clone as detected by CBA [18, 37–39]. The studies describing the significance of multiple unrelated clones with less than 3 chromosome aberrations in the same clone were excluded.

### Literature search

To describe the biological role of CK and its association with other biomarkers in CLL we performed a first search on PubMed using a MeSH controlled vocabulary using the following terms: “Leukemia, Lymphocytic, Chronic, B-Cell” [Mesh] AND “Karyotype” [Mesh] OR “Abnormal Karyotype” [Mesh] OR “Karyotyping” [Mesh]. We found 311 citations without any restriction on publication date. We included in this analysis those paper fulfilling the following requirements: i) English language; ii) biologic characterization including salient clinicobiologic parameters and CBA; iii) single centre or multicentre studies enrolling consecutive patients and studies using a learning cohort and a validation cohort.

We also performed a research on PubMed to identify publications from January 2000 to March 2018 describing the role of the CK as a prognosticator in CLL patients (chemoimmunotherapy and targeted therapy era). The following terms were used: “Leukemia, Lymphocytic, Chronic, B-Cell/drug therapy”[Mesh] AND “Clinical Trial” [Publication Type]. We included in this review only full length manuscripts satisfying these criteria: i) English language; ii) phase 2 or phase 3 clinical trials; iii) multivariate and/or univariate analysis including CK; iv) time to first treatment (TFT), or progression free survival (PFS) or overall survival (OS) as clinical endpoints. Manuscripts describing the prognostic impact of the selected parameters in patients who had received experimental treatment were not included.

## RESULTS

### CK as a consequence of genomic instability

Considering that up to 90% of CLL with CK show an *U-IGHV* mutational status [22–23, 28–29] a relationship may exist between the *IGHV* gene configuration and the development of CK. Indeed a large body of evidence showed that the lymphocytes with *U-IGHV* i) respond to antigen stimulation by activating intracellular signalling, ii) undergo cell divisions *in vivo* as shown by incorporation of deuterated water, iii) carry relatively shorter telomeres and, iv) tend accumulate genomic defects [40–41]. Interestingly, Burns and co-workers [42], used a whole exome sequencing approach to study gene mutations in correlation with the *IGHV* gene configuration and found that exonic CLL driver gene lesions were more common in *U-IGHV* CLL than in CLL with mutated IGHV gene. Coding mutations involved *NOTCH1*, *SF3B1*, *TP53*, *KLHL6* and, less frequently, *IKZF3*, *SAMHD1* and *BIRC3*[42]. These gene mutations may directly increase genome instability reducing the ability of the cells to respond to DNA damage and may also act in an indirect manner, affecting pathways linked to cell proliferation or serving as an important bridge with the microenvironment, which is of particular importance in CLL [43].

Thomay *et al*[44], reported that loss or mutation of *TP53* was associated with an increased number of break events, with frequent involvement of (near-) heterochromatic regions adjacent to the centromeres, generating dicentric chromosomes and whole-arm translocations. In a recent analysis on relapsed/refractory (R/R) CLL, *TP53* mutations preceded clonal evolution leading to the emergence of clones with CK [45]. Furthermore, patients with *TP53* mutations showed significantly shorter telomeres [44, 46–48] a condition causing chromosomal instability [44, 49]. Though few data are available on the association between CK and telomere length, it is worth noting that two studies showed that patients with CK had shorter median telomere length [44, 50]. In patients with 11q-/*ATM* deletions and a CK, the frequency of *TP53* mutations was significantly lower than in patients with CK without del (11q), suggesting that the disruption of the DNA damage control pathway through *ATM* or *TP53* lesions may favour the development of multiple chromosomal rearrangements [44]. Other mutations occurring at a higher incidence in patients with CK involved *FBXW7* (16.7%) in a study [24] and *MYD88* (14.3%) in another study [26]. These genes have been linked to the *NOTCH1/WNT* pathways and to the inflammatory pathway, respectively [51]. *FBXW7* encodes for a tumour suppressive protein, which regulates ubiquitin-mediated degradation of various oncoproteins (cyclin E, c-MYC, NOTCH) [52]. The abnormal binding of cyclin E to *FBXW7* has been related to chromosomal instability in hematopoietic progenitors [53] providing a possible functional link to the development of CK. *MYD88* mutation may have a role in generating genome instability through the activation of the RAS/ERK pathway [54]. Moreover, a recent study by Oliveira-Santos *et al* pointed out a possible role of the histone methyltransferases SET and MYND domain containing 2 (SMYD2) and SET and MYND domain containing 3 (SMYD3), members of the SMYD family of methyltransferases, in the development of CK [55]. In this study, *SMYD2* and *SMYD3* were found to be overexpressed in CLL patients. Interestingly, lower expression of *SMYD2* and *SMYD3* was significantly associated with a CK [55]. Although the mechanism linking these methyltransferases and CK is unknown, it noteworthy that SMYD2 may act as an oncogene by promoting the methylation of p53 and of the retinoblastoma tumor suppressor protein (RB) [56–57], and that SMYD3 promotes MAP3K2 methylation, inducing genomic instability by activation of Ras/Aurora kinase A-driven mechanisms [55, 58–59].

**Figure 1:**
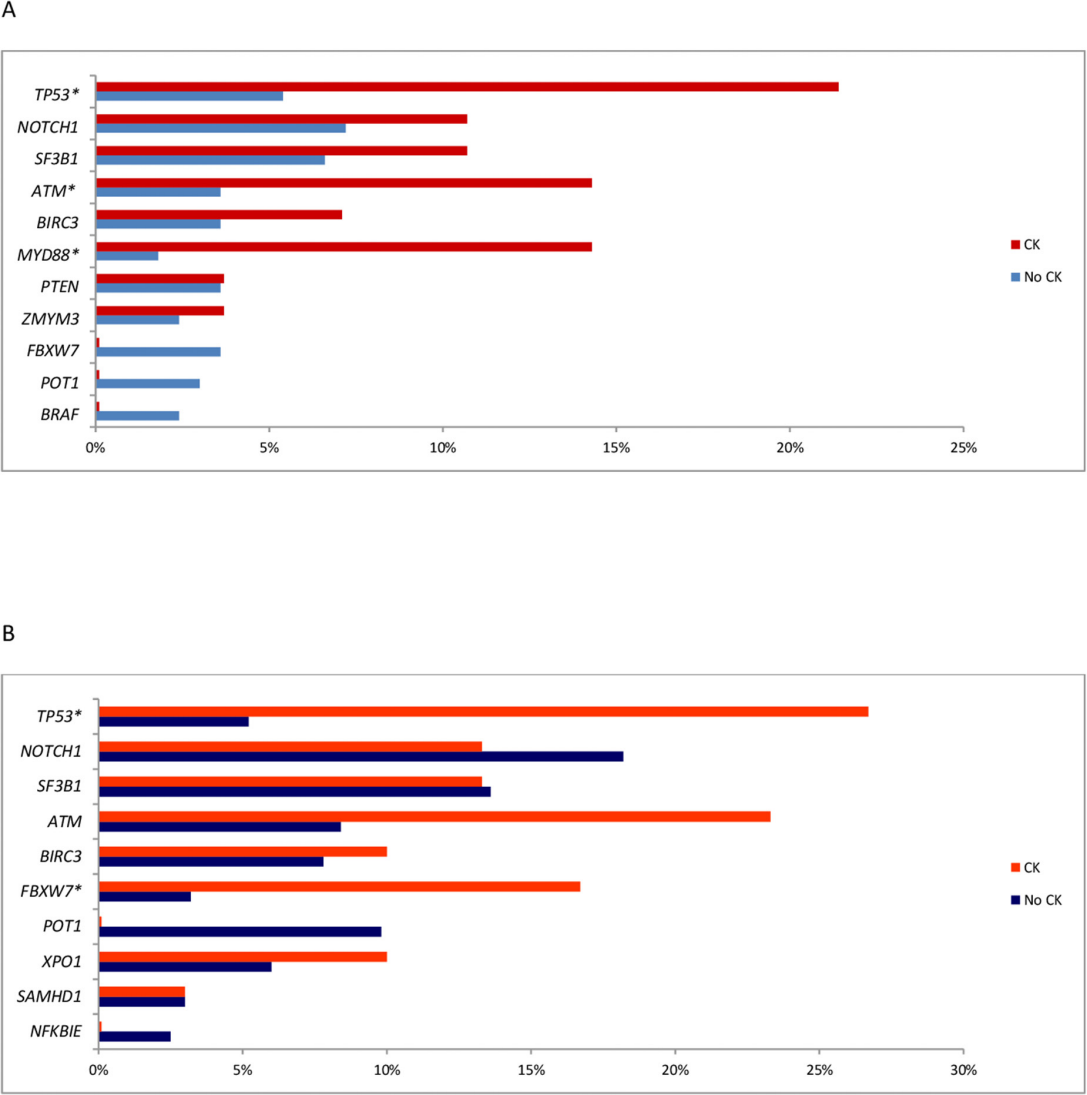
**(A)** Frequency of Gene Mutation by NGS in patient with or without CK reported by Rigolin et al [26] **(B)** Frequency of Gene Mutation by NGS in patient with or without CK reported by Herling et *al* [24]. ^*^p<0.05

**Figure 2:**
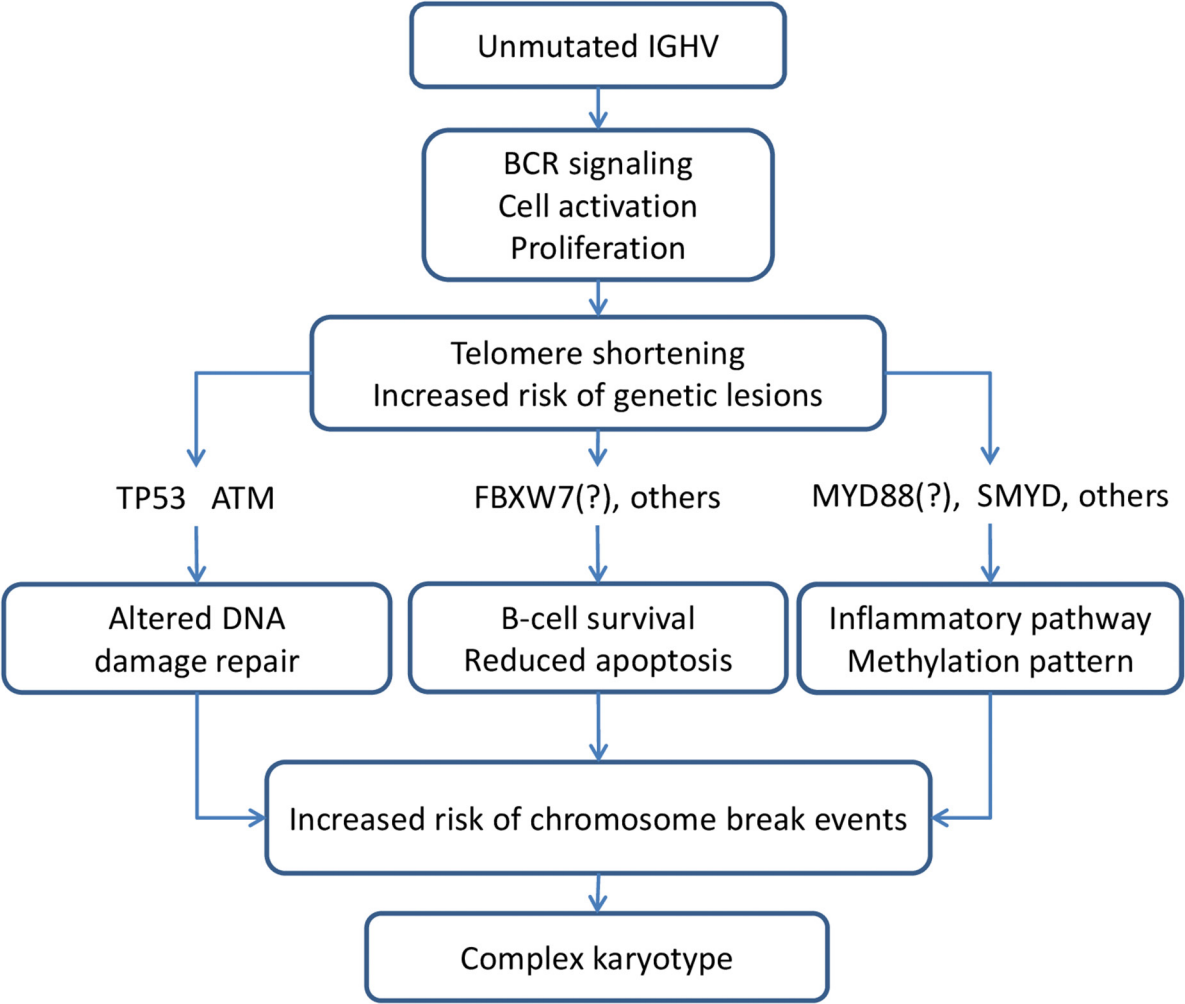
Genetic background favouring the development of complex karyotype

Although the precise mechanisms underlying the development of CK in CLL are have not been elucidated, evidence was provided in 2 studies using CBA and NGS on a panel of CLL driver genes that the CK may be associated with a distinct pattern of genetic lesions (Figure 1). A sequence of events possibly leading to the development of complex cytogenetic rearrangements is illustrated in Figure 2.

### The CK is more frequently found in CLL with unfavorable clinicobiologic features

An extensive biologic characterization of CLL patients enrolled in clinical trials allowed for the identification of biomarkers associated with an inferior prognosis in large international studies recruiting patients treated with chemoimmunotherapy [10, 60]. Because CBA was not included in these studies, we set out analyse possible correlations between CK and clinical parameters (stage, age, sex, performance status) and biomarkers, i.e TP53 disruption, 11q-; unmutated IGHV gene (U-*IGHV*) with a documented prognostic significance at multivariate analysis in the chemoimmunotherapy era [10, 60].

Studies including CBA and the classical clinicobiologic prognosticators showed that no consistent correlation was found between CK and unfavorable clinical parameters, such as age, sex, performance status, beta-2-microglobulin levels, whereas an association appears to be well documented between CK and advanced stage, as shown in Supplementary Table 1.

**Figure 3:**
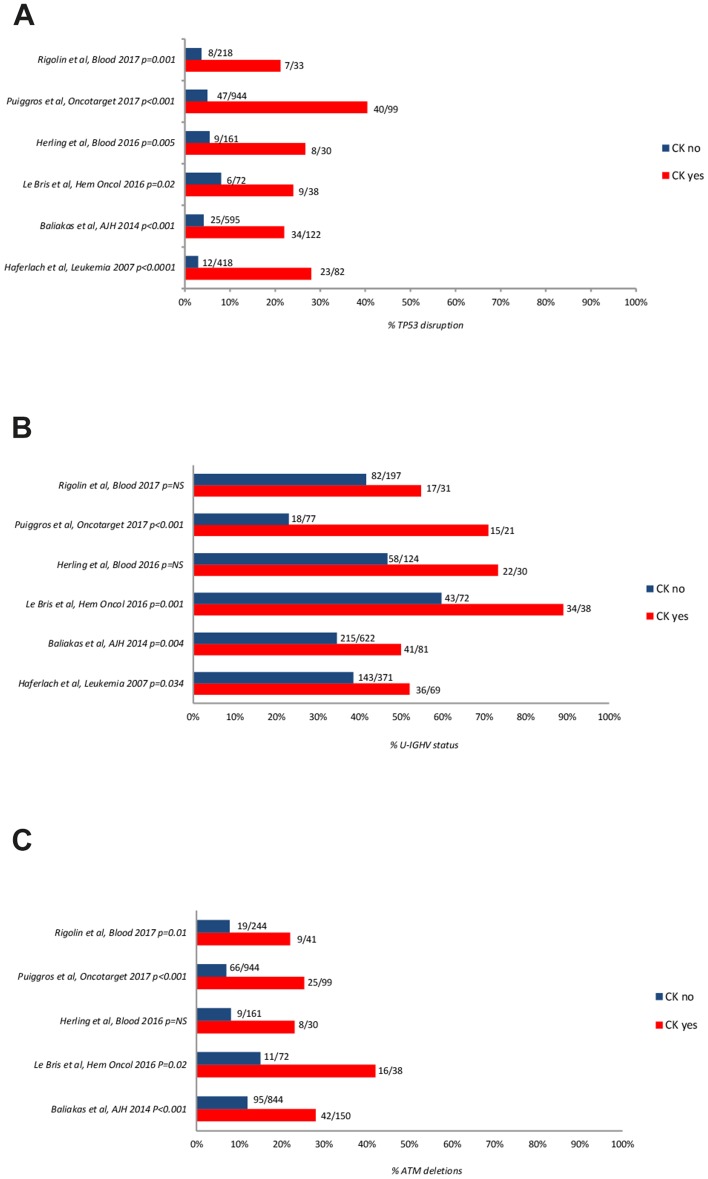
Frequency of *TP53* disruption **(A)**, *ATM* deletion **(B)**, and unmutated *IGHV* gene configuration **(C)** according to the presence or absence of CK. The number of cases are reported aside each bar. NS: not significant; *UM-IGHV*: unmutated IGHV.

The *U-IGHV* status, del (17p)/TP53 mutations or del (11q)/ATM deletions were more frequently seen in CLL with CK, than in CL without CK (Figure 3) [22–24, 28–29, 39, 61–63].

A number of recurrent driver gene mutations was detected in CLL by next generation sequencing (NGS) and excellent reviews highlighted that, while the vast majority of them recur across patients at a low frequency, mutations of *TP53*, *ATM*, *NOTCH1*, and *SF3B1* can be found in >5% of treatment-naïve patients and may predict for an inferior prognosis [48, 64–65]. We therefore analysed available evidence on the association between genetic features and CK. Several studies reported a correlation between CK and *TP53* or *ATM* mutations or the unmutated *IGHV* configuration (Figure 3). The results of 2 studies using CBA with novel mitogens and NGS to detect mutations in large CLL-specific gene panels are shown in Figure 2. Overall, these data show that the incidence of TP53 disruption in CLL with CK was significantly higher (21-80% of the cases) than in an unselected treatment-naïve CLL population (3-13% of the cases) [22–24, 26, 28–29, 63] or in CLL without CK. Likewise, a statistically significant association was found between CK and the unmutated *IGHV* (U-*IGHV*) in the majority of studies (50-90% of the cases) [22–24, 28–29, 63] and with 11q- or *ATM* mutation (22%-42, 1%) (Supplementary Table 1, Figure 1) [23, 26–27, 29, 63]. Herling and colleagues did not find a significant association between CK and the U-*IGHV* status in the patients enrolled in the CLL11 trial [24], possibly due to over-representation of *IGHV*-unmutated cases and consequent low number of *IGHV*-mutated cases in this analysis including patients with disease progression. These findings are not surprising, since *TP53* and *ATM* function are involved in maintaining genomic stability [66–67], and the U-*IGHV* configuration identifies a CLL clone that is responsive to B-cell receptor antigen stimulation with consequent cell activation [40–41].

We also analysed possible association of CK and immunophenotypic markers. In 3 studies the CK was associated with CD38-positivity [23, 63] (Supplementary Table 1). No study analysed whether a CK was associated with CD49d expression, an immunophenotypic marker of unfavorable prognosis [68].

Overall the data here summarized clearly show that there is a strong and reproducible association of CK with advanced stage, *U-IGHV*, TP53 disruption, ATM mutations.

### The CK is a strong adverse risk factor in the chemoimmunotherapy era

The CK was shown to represent an independent adverse prognostic factor in several studies analysing a full set of biomarkers and using robust efficacy endpoints, i.e. time to first treatment (TFT), progression free survival (PFS) or overall survival (OS).

### Impact on TFT

Early observations concerning the negative impact of complex chromosomal abnormalities on clinical outcome date back to the first studies on cytogenetic aberrations in CLL [18–19, 69–71]. In a monocentric study on 109 CLL patients conducted by Mayr *et al*, the CK was found to be predictive of a worse TFT at univariate analysis (median TFT, 26 months; 95% CI, 15-37 months vs 106 months 95% CI, 61-151 months; P < 0.001) [20]. In another monocentric study on 482 CLL patients [38], the CK was associated with a shorter TFT in treatment-naïve patients at univariate analysis (HR 1.644; P = 0.029) and similar findings were reported by Travella and coworkers, who observed a 13-month median TFT in patients with CK as compared with 69-month median TFT in patients without CK (P=0.015) [72]. These observations were also reported in a larger study on 1001 previously untreated CLL where the CK was associated with a shorter TFT at univariate (p=0.01) and multivariate analysis [23]. In subsequent studies the prognostic impact of CK on TFT was documented in a learning cohort (LC) including 166 patients and in an independent validation cohort (VC) including 250 patients (HR 5.004; 95% CI, 1.980-12.597; P<0.001 and HR 2.159; 95% CI, 1.499-5.331; P<0.001, respectively). The difference held at multivariate analysis in the LC (HR 2.418; 95% CI; 1.173–4.983; P=0.017) [39]. More recently, Puiggros *et al*, conducted a study on 1045 untreated MBL/CLL patients to evaluate the clinical impact of CK and high risk FISH (HR-FISH) on outcome [63]. The cases with CK had a higher 2-year cumulative incidence of progression requiring treatment (48%; 95% CI, 36-58% vs. 20%; 95%CI, 18-23%; P<0.001) [63]. Finally, in a large retrospective monocentric study by Rigolin *et al* including 335 newly diagnosed CLL, the CK was associated with a shorter TFT, independent from CLL-IPI (HR 2.157; IC 95%, 1.177-3.952; 0.013) [27].

### Impact on PFS

The correlation between CK and PFS was investigated in few clinical trials in the era of chemoimmunotherapy (CIT). In a study by Badoux *et al*, the CK proved to be predictive of shorter PFS in 284 R/R CLL patients treated with FCR (HR 2.6; 95%CI, 1.5-4.4; P<0.001) [73]. Similar data were presented in another study including 80 R/R CLL patients treated with Cyclophosphamide, Fludarabine, Alemtuzumab, and Rituximab [74]. In this analysis the CK along with del (17p) were significantly associated with worse PFS (HR 4.1; 95%CI, 2.0-8.4; P<0.001). Likewise a retrospective study on 110 CLL patients treated with first-line FCR showed that the CK was associated with shorter median PFS (21 vs. 55 months; HR 2.4; 95%CI, 1.14-5.19; P=0.002) [29]. Herling and coworkers described the impact on outcome of CK in a subset of patients treated with chlorambucil-based chemo- or chemoimmunotherapy in the CLL11 trial and found that chromosome translocations, a type of chromosome imbalance often associated with complex karyotype [75], was associated with shorter PFS [24].

### Impact on OS

As shown in Table 1, a significantly shorter survival was observed in virtually all the observational studies that included treatment naïve patients, including a monocentric study on CLL patients treated with first-line FCR [29] and an analysis using a learning cohort and a validation cohort [39]. Interestingly, the presence of CK was shown to represent an adverse prognostic parameter that was independent of the international prognostic index [27].

**Table 1:** Impact of complex karyotype on OS

	Tot. pts	CK	Treatment	Disease status	Median OS (months)		Univariate analysis		Multivariate analysis	
*Reference*					CK yes	CK no	HR (IC 95%)	P	HR (IC 95%)	P
[20]	109	NA	various	TN and pretreated	107	346	15.44^*^	<0.001	-	Ø
[38]	482	71/399 (17.8%)	NA	TN	81.1% at 5y	86-94.4% at 5y #	3.830 (1.714-8.560)	0.001	-	Ø
[73]	284	22/182 (12.1%)	FCR	R/R	26	10.5 m. -78 m. ##	**-**	**-**	1.9 (1.1-3.2)	0.015
[74]	80	8/67 (11.9%)	CFAR	R/R	NA	NA	**-**	**-**	2.0 (1.1-3.7)^**^	0.022
[72]	38	16/38 (42.1%)	NA	TN	56	144	**-**	**-**	-	-
[39]	LC 166 VC 250	20/145 (13.8%) 35/238 (14.7%)	various	TN	NA	NA	2.701 (1.988-8.787) 2.155 (1.160-4.004)	<0.001 0.015	4.856 (1.475-9.998) 3.630 (1.358–9.703)	<0.0001 0.010
[29]	110	38/110 (34.5%)	FCR	TN	72.4% at 5 y	85.8% at 5y	**-**	**-**	5.16 (1.2-22.1)	0.07
[24]	161	30/154 (19.5%)	Clb/Clb-R/Clb-G	TN	NA	NA	2.9 (1.5-5.4)° 2.6 (1.3-5.4)°°	0.001° 0.006°°	2.682 (1.366-5.264)	0.004
[63]*^#^*	1045	99/1043 (9.5%)	various	TN	79	NR	**-**	**-**	1.66 (1.06-2.59)	0.027
[27]	335	41/287 (14.3%)	various	TN	70	135	3.176 (1.882-5.359)	<0.001	3.572 (1.341-9.515)	0.011
[77]	186	37/186 (19.8%)	Lenalidomide-R Lenalidomide-O	R/R	23	62.8	-	-	2.08 (1.15-3.76)	0.015

TN=treatment naïve; R/R= relapsed/refractory; OS=overall survival; CK=complex karyotype; NA=not available; Clb=chlorambucil; FCR=Fludarabine, Cyclophosphamide, Rituximab; G=Obinutuzumab; R= Rituximab; O-Ofatumumab; Ø=not significant; LC: learning cohort; VC: validation cohort

^*^log rank; ^**^CK or 17p aberration; °all arms; °°(Clb-R+Clb-G);

^#^MBL/CLL

# OS was compared in this study between patients with CK and patients without CK carrying chromosome translocations, or 1-2 aberrations or normal karyotype.

## OS in the patients without CK was reported in this study according to the aberration detected by FISH (13q-, +12, 11q-, 17p).

In 2016, Herling *et al*[24], performed an analysis in a subgroup of 161 elderly and comorbid patients enrolled in the CLL11 trial comparing the efficacy of chlorambucil alone, against chlorambucil with rituximab or with the second generation anti CD20 obinutuzumab. The patients with available karyotype included in analysis were representative of the entire study cohort with the exception of a higher percentage of patients >70 years. A CK in 30/154 (19.5%) patients was shown to represent an independent negative prognostic factor on OS along with advanced stage, elevated beta-2-microglobulin (β2M) an unmutated *IGHV* gene and mutations in the *POT1* gene. Interestingly, in a phase II trial evaluating chlorambucil and rituximab in 85 treatment naïve patients [76] the presence of CK was associated with a lower overall response rate (ORR) and complete remission rate, which represent surrogate endpoints of OS. Indeed Takahashi *et al*, more recently confirmed the adverse impact of CK on ORR and OS in a study conducted on 186 R/R CLL patients treated with a Lenalidomide based regimen [77]. Finally, in two trials including relapsed/refractory CLL, the CK was significantly associated with a shorter OS in patients treated with fludarabine cyclophosphamide and rituximab, with or without alemtuzumab [73–74].

### CK is an independent adverse prognosticator in high risk CLL

A negative prognostic impact of CK was recently documented even when the analysis was restricted to high risk CLL as defined by unfavorable genetic features [23–24, 28, 78]. Herling *et al*, showed that patients with CK alone exhibited a similar survival as those with *TP53* lesion, whereas patients with both CK and *TP53* aberrations had a particularly poor prognosis (P<0.001) [24]. In another study on 101 patients carrying *TP53* abnormalities, 31/101 cases (47%) showed a CK. CK was associated with shorter OS at multivariate analysis (HR 2.47; 95% CI, 1.11–5.49; P=0.027) confirming that CK may portend a dismal outcome in this aggressive subgroup [78].

In a study on previously untreated high-risk CLL (defined by the presence of U-*IGHV* gene and/or chromosome 11q22 deletion and/or chromosome 17p13 deletion and/or *TP53* mutations) the presence of CK in 21/101 (20.8%) cases has been associated with unfavorable FISH (i.e. 11q- or 17p-) (P<0.001) and *TP53* disruption (P=0.012). In multivariate analysis, the CK was significantly associated with a shorter TFT (HR 2.934; 95% CI, 1.686-5.108; P<0.001), an inferior OS (HR 2.914; 95% CI, 1.080-7.861; P = 0.024) and a trend toward a shorter time to chemorefractoriness (TTCR) (HR 2.486; 95% CI, 0.905-6.825; P=0.077) [28].

In the subgroup of patients with concurrent *TP53* disruption and unfavorable karyotype, the median TFT reported in this study was 5.5 months compared to 97.3 months in patients with sole *TP53* disruption (p<0.0001). Likewise, the median OS and TTCR, were much shorter in this subgroup (28.7 months and 15 months, respectively) than in patients with sole *TP53* disruption who did not reach the median OS (P<0.0001) and showed a TTCR of 30 months (P<0.0001) [28].

Similar observations were reported by Baliakas *et al*, who found a trend for a significantly shorter TFT in patients with del(17p) and concomitant CK as compared with patients with del(17p) alone (P=0.06) [23].

Finally, Le Bris *et al*, reported a dismal outcome in patients with concurrent *TP53* disruption, U-*IGHV* and CK, with a shorter PFS (12 vs. 55 months; HR: 8.1, 95% CI 1.13–57.39; P<0.0001) and shorter OS (5–year OS: 21.3 ± 18% vs 84.7 ± 8%; HR: 25.7, 95% CI 1.15–574; P < 0.0001) as compared with the patients without this combination of adverse risk factors [29].

### The CK may represent an unfavorable prognostic marker in the targeted therapy era

Whereas mechanism-based treatment with novel agents proved very efficacious in unfavorable genetic subsets of CLL (i.e. U-IGHV, TP53 disruption, 11q-) [79–81] several studies showed that the CK may have a negative prognostic role in R/R CLL receiving kinase targeted treatment or the BCL2 inhibitor venetoclax, as summarized in Table 2 [14, 61, 79, 82–83]. A retrospective analysis on 88 R/R CLL patients receiving Ibrutinib based regimens demonstrated that the CK was a stronger predictor of an inferior outcome than del(17p) [61]. A CK and del(17p) were found in 21/56 (38%) and 34/86 (40%) assessable cases, respectively, whereas they coexisted in 17 cases. At a median follow-up for surviving patients of 28 months (range, 14-48 months) the CK was associated with a shorter event free survival (EFS) (HR 6.6; 95% CI, 1.7-25.6; P = 0.006) and inferior OS (HR 5.9; 95% CI, 1.6-22.2; P = 0.008). Furthermore, a trend for a shorter EFS was observed in patients with a CK and del(17p) vs. those with sole del(17p) (P = 0.056), whereas no association was noted between del(17p) and OS (P = 0.885) [61]. It is worth noting that *TP53* mutations were not assessed in this analysis. Brown *et al*, analysed the prognostic significance of CK in the phase 3 RESONATE study, including 195 R/R CLL patients treated with Ibrutinib [14]. At a median follow up of 19 months the presence of CK in 39/153 (25%) assessable patients did not show a significant impact on PFS (HR 1.53, 95% CI, 0.741-3.157; P=0.2476) and on OS (HR 1.86; 95% CI, 0.770-4.485; P=0.1610). It is noteworthy that, at a 5-year follow-up of phase-2 studies, the presence of CK was associated with a highly significant difference in median PFS in R/R CLL (31 months with CK compared to not reached in patients without CK and with 51 months in the entire cohort). However, the only genetic parameter retaining its adverse significance at multivariable analysis in this study was represented by the 17p deletion [79]. The CK and/or del(17p) may predispose to ibrutinib resistance through the development of the BTK C481S mutation or phospholipase Cγ2 (PLCγ2) activating mutation [61, 84–86].

**Table 2:** Impact of complex karyotype on R/R CLL patients treated with pathway inhibitors

Therapy and patient population	Tot. pts	CK	Median OS (months)		OS		Median PFS (months)		PFS	
			CK yes	CK no	HR (IC 95%)	P	CK yes	CK no	HR (IC 95%)	P
*Ibrutinib in R/R CL* [14, 61]	88	21/56 (37.5%)	25	NR	5.9 (1.6-22.2)	0.008	19	39	6.6 (1.7-25.6)^*^	0.006
	195	39/153 (25.5%)	NR	NR	1.86 (0.770-4.485)	0.161	NR	NR	1.53 (0.741-3.157)	0.248
*Idelalisib in R/R CLL* [82]	110	26/65 (40%)	NR	NR	1.78 (0.69-4.64)	0.230	20.9	19.4	1.18	0.630
*Venetoclax in R/R CLL post KI* [83]	67	16/38 (42.1%)	-	-	-	-	16	NR	6.6 (1.5-29.8)^**^	0.005

R/R relapsed/refractory; OS = overall survival; TFT = time to first treatment; EFS = event free survival; PFS = progression free survival; NR = not reached; ^*^Event Free Surival (EFS); ^**^Time to progression (TTP).

The CK was shown to have a strong adverse impact on outcome in patients treated with Venetoclax [83]. Among 67 R/R CLL patients treated with this BCL-2 inhibitor, 16 out of 38 (42%) assessable patients had CK. In univariate analysis the CK was associated with higher risk of progression (HR 6.6; 95% CI, 1.5-29.8; P = 0.005) along with fludarabine-refractory status (HR 7.01; 95%CI, 1.7-28.5; P = 0.002). Multivariate analysis was not performed due to the small sample size in this study. Interestingly, the presence of a CK increased the risk of progression among patients with F-refractory disease (P = 0.002), whereas TP53 mutation and/or del(17p) did not show any impact on time to progression (TTP) [83].

In patients treated with idelalisib and rituximab, the prognosis was not significantly influenced by the presence of a CK in the analysis by Kreuzer and coworkers, who reported their experience on 65 patients with available karyotype drawn from 110 R/R CLL patients receiving Idelalisib plus rituximab. With a relatively short follow-up (median of 21.4 months), 26 patients with CK showed no significant difference in terms of PFS and OS as compared with 39 patients without CK [82].

Prospective studies are needed to support the circumstantial evidence summarized here that a CK may represent a prognosticator in patients with R/R CLL receiving new oral agents.

### CK and Richter transformation

The presence of CK was sporadically linked to the development of RT in previous reports [87–89]. In a retrospective study on CLL patients treated with first-line FCR, Le Bris *et al*, reported a CK in 1/4 cases with RT [29]. Anderson *et al* found a CK in 12 of 25 patients (48%) with progression on Venetoclax, including 8 of 17 patients with RT (47%) [83]. Rogers *et al*, studied the impact of CK in 46 CLL patients who developed RT. They reported a CK in 28/42 (67%) patients who subsequently developed RT and found that CK had an adverse impact at multivariate analysis on OS with the R-EPOCH regimen (HR 2.72; CI 95%, 1.14-6.52; P=0.025) [90].

In a recent analysis, Miller *et al*, found an association between near-tetraploidy (4 copies of most chromosomes within a cell) with CK and showed that 6/9 patients with this peculiar cytogenetic pattern developed RT. In a multivariate analysis near-terapolidy and CK represented independent predictors of ibrutinib discontinuation due to transformation [91].

Further studies are required to define the correlation between CK and RT

### Perspectives

The importance of cytogenetic analysis in CLL was recognized by the 2018 guidelines of the International Workshop on CLL [92], which proposed the introduction of CBA in future prospective clinical trials to validate the prognostic and predictive value of karyotype aberrations [93]. The following issues represent an area of investigation which may facilitate the introduction of cytogenetic analysis in clinical practice.

### Refinement of the definition of CK

Evidence was provided that this broad cytogenetic category, defined by the presence of at least 3 chromosome aberrations, should be regarded as heterogeneous. The patients with CK due to the coexistence of trisomy of chromosomes 12, 18 and 19, showed favourable clinicobiologic characteristics in terms of age (median 59 years), high incidence of mutated *IGHV* status and low frequency of *TP53* disruption or *NOTCH1* mutation (5% and 4% of cases, respectively) [23, 94, 95]. Likewise, Baliakas and colleagues identified a subset of patients with CK carrying +12, +19 plus other numerical and/or structural chromosome abnormalities (12% of the cases), which displayed indolent clinical course independent of clinical stage, *IGHV* mutational status and *TP53* status [96]. These findings suggest that the cytogenetic complexity defined solely by numerical aberrations should not be regarded as an unfavorable prognostic marker in CLL. Recently, Rigolin *et al*, showed that within patients carrying CK as defined by the presence of 3 or more aberrations, the presence of unbalanced translocations (i.e. chromosome additions, derivatives, insertions, duplications, ring-, dicentric- and marker-chromosomes) was associated with a worse outcome in terms of OS and TTFT (HR 2.773; 95% CI, 1.056-7.281; P=0.038 and HR 2.375; 95% CI, 1.027-5.492; P=0.043) [97]. Interestingly, a distinct mRNA expression profile, with a deregulation of genes involved in cell cycle control and DNA damage response, was documented in patients with a CK carrying unbalanced rearrangements [97].

Finally, of the presence of ≥5 chromosomal aberrations, referred to as high-CK, predicted for a particularly aggressive clinical course in a large multicentre study, possibly due to a strong association with *TP53* disruptions (P<0.001) [23]. More recently high-CK (i.e. ≥5 chromosomal aberrations) was shown to represent a strong adverse prognosticator independent of clinical stage, *IGHV* mutational status and *TP53* status [96].

### New methods of detection

CBA is somewhat laborious, has a low sensitivity and requires mitotic stimulation of fresh or frozen living cells. Hence, alternative methods of detection of genomic complexity were developed. The array-based (CGH/SNP) analysis offers the opportunity to study the CLL genome, does not require *in vitro* mitogens and allows for the detection of subtle DNA gains and losses [98]. Leeksma *et* al, recently analysed 1911 patients with monoclonal B-cell lymphocytosis and treatment-naïve CLL and found that 451/1911 cases (24%) displayed genome complexity (defined as the presence of ≥3 structural and/or numerical aberrations) [99]. This study also showed that array-analysis detected more aberrations than CBA (2.35 vs 1.84, 95% CI paired differences 0.221-0.798) [99].

Diagnostic platforms using whole genome sequencing (WGS) to detect single nucleotide variants and insertion/deletions are being developed and validated for potential usage in clinical practice [100]. Although these methods will likely provide comprehensive genomic characterisation of CLL and will represent alternative method to recognize the prognostic or predictive role genetic lesions in trials, they still require standardization and a univocal definition of “genome complexity”.

At the moment CBA represents a standardized tool for risk assessment in CLL, providing complementary information to FISH and traditional genetic studies of recurrent mutations. Because cytogenetic laboratories are available in the majority of hematologic centres and the mitotic yield greatly improved with novel mitogens, CBA could be incorporated in prospective trials to definitely establish its predictive power in an era in which both CIT and new mechanism-based treatment are available [92].

## SUPPLEMENTARY MATERIALS FIGURES AND TABLES


