# Supplementary material for: Biological significance and prognostic/predictive impact of complex karyotype in chronic lymphocytic leukemia
Source: Oncotarget. 2018 Sep 28;9(76):34398–412. doi: 10.18632/oncotarget.26146 (PMC6188145; doi:10.18632/oncotarget.26146)
Supplement: Supplementary file 1 [file oncotarget-09-34398-s001.pdf]

Biological significance and prognostic/predictive impact of complex karyotype in chronic lymphocytic leukemia

SUPPLEMENTARY MATERIALS

Supplementary Table 1: Association between complex karyotype, gene mutations and other biomarkers^

Reference	+N. pts	CK	Binet B/C	P	*17p-/ TP53^{MUT}*	P	*11q-/ ATM^{MUT}*	P	*FBXW7^{MUT}*	P	*MYD88^{MUT}*	P	*U-IGHV*	*P*	*CD38+*	P
[22]	n=500	n=82 (16.4%)	-	-	23/82 (28%)	<0.0001	-	-	-	-	-	-	36/69 (52.2%)**	0.034	42/68 (61.8%)**	0.02
[23]	n=1001	n=157 (15.7%)	28/112 (22.9%)**	0.05	34/122 (28%)**	<0.001	42/150 (28%)**	<0.001	-	-	-	-	41/81 (50%)**	0.004	-	-
[29]	n= 110	n=38 (34.5%)	-	-	(9/38) 24%	0.02	16/38 (42%)	0.02	-	-	1/38 (2.6%)	Ø	34/38 (89%)	0.001	-	-
[24]	n=154	n=30 (19.5%)	-	-	8/30 (26.7%)	0.005	7/30 (23.3%)	Ø	5/30 (16.7%)	0.025	-	Ø	22/30 (73.3%)	Ø	-	-
[26]	n=195	n=28 (14.4%)	-	-	6/28 (21.4%)	0.003	4/28 (14.3%)	0.018	0/28 (0%)	Ø	4/28 (14.3%)	0.001	-	-	-	-
[63]§	n=1043	n=99 (9.5%)	22/99 (22%)	0.001	40/99 (40.4%)	<0.001	25/99 (25.3%)	<0.001	-	-	-	-	15/21 (71%)**	<0.001	33/99 (33%)	0.007
[28]	n=101	n=21 (20.8%)	7/21 (33.3%)	Ø	8/21 (38.1%)	0.021	4/21 (19%)	Ø	-	-	-	-	19/21 (90.4%)	Ø	14/21 (66.7%)	Ø
[27]	n=287	n=41 (14.3%)	13/41 (31.7%)	0.013	7/33 (21.2%)**	0.001	9/41 (21.9%)	0.01	-	-	-	-	17/31 (54.8%)**	Ø	25/41 (61%)	0.003

^ the p values refer to the frequency of each biomarker in the subgroup of patients with CK as compared with patients without CK
+ N. of patients assessed by conventional banding analysis
§ MBL or CLL patients
**data available for a subgroup of patients
Ø = not significant
